# 4-Chloro­benzoyl-*meso*-octa­methyl­calix[2]pyrrolidino[2]pyrrole: an acyl chloride derivative of a partially reduced calix[4] pyrrole

**DOI:** 10.1107/S1600536812007003

**Published:** 2012-03-07

**Authors:** Guillaume Journot, Reinhard Neier, Helen Stoeckli-Evans

**Affiliations:** a Institute of Chemistry, University of Neuchâtel, Avenue de Bellevaux 51, CH-2000 Neuchâtel, Switzerland; b Institute of Physics, University of Neuchâtel, Rue Emile-Argand 11, CH-2000 Neuchâtel, Switzerland

## Abstract

In the title compound, C_35_H_47_ClN_4_O, the two pyrrolidine rings have envelope conformations. The conformation of the macrocycle is stabilized by N—H⋯N hydrogen bonds and a C—H⋯N inter­action. The benzoyl ring is inclined to the adjacent pyrrole ring by 11.66 (11)°, with a centroid–centroid distance of 3.7488 (13) Å. In the crystal, molecules are linked by N—H⋯O hydrogen bonds into helical chains propagating in [010] and C—H⋯O and C—H⋯π interactions are also observed.

## Related literature

For the heterogeneous catalytic hydrogenation of *meso*-octa­methyl­calix[4]pyrrole, which gave *meso*-octa­methyl­calix[2]pyrrole­[2]pyrrolidine, see: Blangy *et al.* (2009 ▶). For *N*-acyl­ation of pyrrolidines, using substituted benzoyl chlorides, see: Journot *et al.* (2012*a* ▶); Zhang *et al.* (2009 ▶). For the synthesis and reactivity of the title compound, see: Journot & Neier (2012 ▶). For the crystal structures of similar compounds, see: Journot *et al.* (2012*b* ▶,*c* ▶,*d* ▶,*e* ▶).
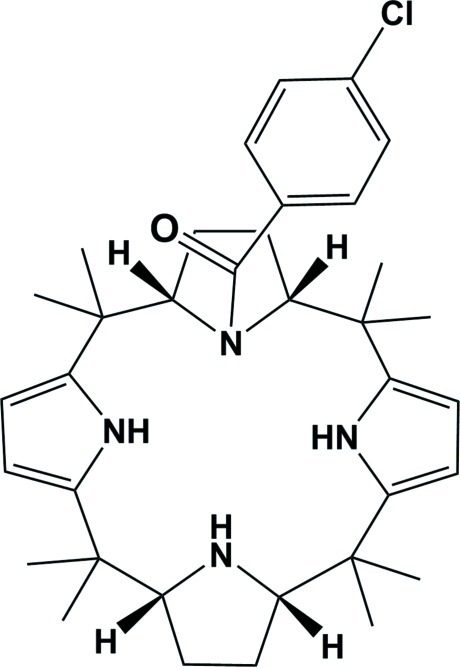


## Experimental

### 

#### Crystal data

C_35_H_47_ClN_4_O
*M* *_r_* = 575.22
Monoclinic, 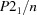

*a* = 10.3224 (6) Å
*b* = 12.0389 (4) Å
*c* = 25.3311 (13) Å
β = 96.798 (4)°
*V* = 3125.8 (3) Å^3^
*Z* = 4
Mo *K*α radiation
μ = 0.16 mm^−1^
*T* = 173 K
0.45 × 0.42 × 0.40 mm

#### Data collection

Stoe IPDS 2 diffractometer
Absorption correction: multi-scan (MULscanABS in *PLATON*; Spek, 2009 ▶) *T*_min_ = 0.973, *T*_max_ = 1.000
32665 measured reflections
5906 independent reflections
4215 reflections with *I* > 2σ(*I*)
*R*_int_ = 0.076

#### Refinement

*R*[*F* ^2^ > 2σ(*F* ^2^)] = 0.053
*wR*(*F* ^2^) = 0.102
*S* = 1.03
5906 reflections
381 parameters
1 restraint
H atoms treated by a mixture of independent and constrained refinement
Δρ_max_ = 0.24 e Å^−3^
Δρ_min_ = −0.34 e Å^−3^

### 

Data collection: *X-AREA* (Stoe & Cie, 2009 ▶); cell refinement: *X-AREA*; data reduction: *X-RED32* (Stoe & Cie, 2009 ▶); program(s) used to solve structure: *SHELXS97* (Sheldrick, 2008 ▶); program(s) used to refine structure: *SHELXL97* (Sheldrick, 2008 ▶); molecular graphics: *PLATON* (Spek, 2009 ▶); software used to prepare material for publication: *SHELXL97*, *PLATON* and *publCIF* (Westrip, 2010 ▶).

## Supplementary Material

Crystal structure: contains datablock(s) I, global. DOI: 10.1107/S1600536812007003/zq2154sup1.cif

Structure factors: contains datablock(s) I. DOI: 10.1107/S1600536812007003/zq2154Isup2.hkl

Supplementary material file. DOI: 10.1107/S1600536812007003/zq2154Isup3.cml

Additional supplementary materials:  crystallographic information; 3D view; checkCIF report

## Figures and Tables

**Table 1:** Hydrogen-bond geometry (Å, °) *Cg*1 is the centroid of pyrrole ring N2/C3/C4/C25/C26; *Cg*2 is the centroid of the benzene ring C30–C35.

*D*—H⋯*A*	*D*—H	H⋯*A*	*D*⋯*A*	*D*—H⋯*A*
N2—H2⋯N3	0.88	2.31	2.865 (2)	121
N4—H4⋯N3	0.88	2.55	3.051 (2)	117
C15—H15*A*⋯N2	0.98	2.52	3.488 (3)	171
C15—H15*A*⋯*Cg*1	0.98	2.40	3.301 (2)	152
N3—H3*N*⋯O1^i^	0.882 (18)	2.257 (18)	3.105 (2)	161.1 (18)
C23—H23*B*⋯O1^i^	0.98	2.53	3.495 (3)	168
C27—H27*C*⋯*Cg*2^i^	0.98	2.82	3.702 (2)	150

Symmetry code: (i) 
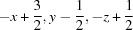
.

**Fig. 1.**
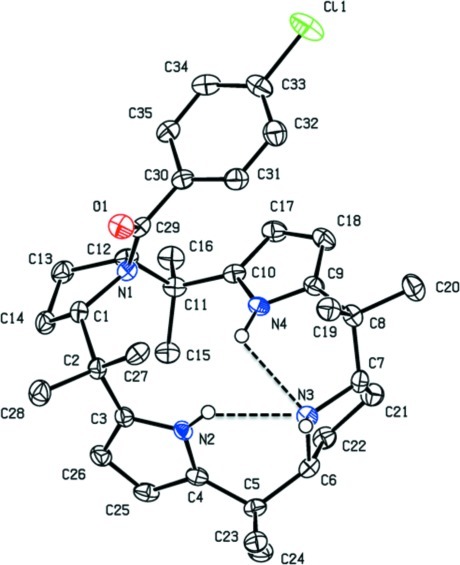
A view of the molecular structure of the title compound, with the numbering scheme and displacement ellipsoids drawn at the 50% probability level. The N—H···N hydrogen bonds are shown as dashed lines (see Table 1 for details; the C-bound H atoms have been omitted for clarity).

**Fig. 2.**
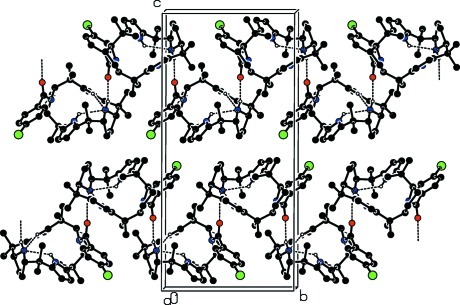
A view along the a axis of the crystal packing of the title compound. The N—H···N and N—H···O hydrogen bonds are shown as dashed lines (see Table 1 for details; the C-bound H atoms have been omitted for clarity).

**Fig. 3.**
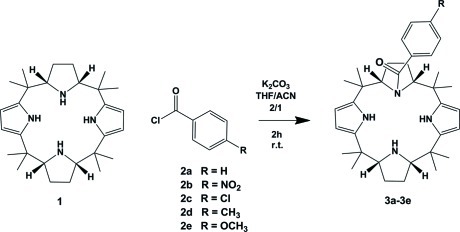
The general procedure for the N-acylation of meso-octamethylcalix[2]pyrrolidino[2]pyrrole (1).

## supplementary crystallographic information

### Comment

We have recently reported the access to new macrocycles by heterogeneous catalytic hydrogenation of *meso*-octamethylcalix[4]pyrrole, which gave *meso*-octamethylcalix[2]pyrrole[2]pyrrolidine (1 in Fig. 3) [Blangy *et al.*, 2009]. It was decided to investigate the nucleophilicity of this new macrocycle, which showed interesting reactivity (Journot & Neier, 2012), by reacting different substituted benzoyl chlorides with the macrocycle under smooth conditions [Journot *et al.*, 2012*a*; Zhang *et al.*, 2009]. Herein, we report on the synthesis and crystal structure of the title 4-chlorobenzoyl derivative, one of five compounds that have been studied by X-ray diffraction analysis (Journot *et al.*, 2012*b*,*c*,*d*,*e*).

The molecular structure of the title compound is given in Fig. 1. The two pyrrolidine rings (N1,C1,C12—C14) and (N3,C6,C7,C21,C22) have envelope conformations with, respectively, atoms C13 and C6 as the flaps. The conformation of the macrocycle is stabilized by intramolecular N—H···N hydrogen bonds involving atom N3 and the two pyrrole H atoms, H2 and H4 (Fig. 1 and Table 1). The benzoyl ring (C30—C35) is inclined to the pyrrole ring (N4,C9,C10,C17,C18) by 11.66 (11)°, with a centroid-to-centroid distance of 3.7488 (13) Å. The methyl group C15 is also in close contact with the pyrrole ring (N2,C3,C4,C25,C26), with a short C15—H15A···N2 interaction and a C15—H15A···centroid distance of 3.301 (2) [see Table 1].

In the crystal, molecules are linked *via* an N—H···O hydrogen bond, involving the N3 pyrrolidine H-atom (H3N) and the benzoyl O atom (O1), leading to the formation of helical chains propagating along [010] - see Fig. 2 and Table 1. The same O atom is involved in a C—H···O contact with methyl group C23. A C—H···π interaction is also observed, involving the methyl group C27 and the benzoyl ring (C30—C35) [see Table 1].

The overall geometry and crystal packing is very similar to that reported for the 4-methoxybenzoyl derivative (Journot *et al.*, 2012*b*), and the 4-nitrobenzoyl (Journot *et al.*, 2012*d*) and 4-methylbenzoyl (Journot *et al.*, 2012*e*) derivatives. The benzoyl derivative (Journot *et al.*, 2012*c*) crystallized, in the trigonal space group *R*-3, as a partial (0.25H_2_O) hydrate, and forms hydrogen bonded chains propagating along [001].

Footnote to Table 1: *Cg*1 is the centroid of pyrrole ring (N2,C3,C4,C25,C26); *Cg*2 is the centroid of the benzene ring (C30—C35).

### Experimental

The general procedure for the *N*-acylation of *meso*-octamethylcalix[2]pyrrolidino[2]pyrrole (1) is illustrated in Fig. 3. A two-necked flask fitted with a gas inlet and containing a stirrer bar was charged with 100 mg (0.23 mmol) of *meso*-octamethylcalix[2]pyrrolidino[2]pyrrole (1), 4-chlorobenzoyl chloride (2c) (53.61 µ*L*, 0.48 mmol), potassium carbonate (70 mg, 0.48 mmol) in THF (5 ml) and acetonitrile (2.5 ml). The reaction vessel was flushed with argon and sealed with a septum. After 15 min. a precipitate appeared and the reaction mixture was stirred for 2 h room temperature. 10% sodium carbonate was then added and the reaction mixture was extracted with dichloromethane. The organic layer was washed successively with two × 10% sodium carbonate and saturated brine. The organic layer was dried with sodium sulfate, and the solvents were removed under vacuum. The residue was purified by column chromatography (SiO_2_, CH_2_Cl_2_/MeOH, 97/3) to yield 131.6 mg (75.6%) of colourless crystals of the title compound (3c). Melting point: 501 K. HRMS calcd. for C_35_H_47_ClN_4_O^+^H^+^ 575.3511; found 575.3510. Further synthetic and spectroscopic data have been reported elsewhere (Journot & Neier, 2012).

### Refinement

The NH H-atoms were located in a difference electron-density map. H-atom H3N was freely refined while the other NH H-atoms and the C-bound H-atoms were included in calculated positions and treated as riding atoms: N—H = 0.88 Å, C—H = 0.95 Å for CH-allyl and CH-aromatic H atoms, and 1.00, 0.99 and 0.98 Å, for methine, methylene and methyl H atoms, respectively, with *U*_iso_(H) = k × *U*_eq_(N,*C*), where k = 1.5 for CH_3_ H-atoms, and 1.2 for the other H-atoms.

### Crystal data

**Table:**

C_35_H_47_ClN_4_O	*F*(000) = 1240
*M**_r_* = 575.22	*D*_x_ = 1.222 Mg m^−^^3^
Monoclinic, *P*2_1_/*n*	Melting point: 501 K
Hall symbol: -P 2yn	Mo *K*α radiation, λ = 0.71073 Å
*a* = 10.3224 (6) Å	Cell parameters from 16706 reflections
*b* = 12.0389 (4) Å	θ = 1.6–26.1°
*c* = 25.3311 (13) Å	µ = 0.16 mm^−^^1^
β = 96.798 (4)°	*T* = 173 K
*V* = 3125.8 (3) Å^3^	Block, colourless
*Z* = 4	0.45 × 0.42 × 0.40 mm

### Data collection

**Table:**

Stoe IPDS 2 diffractometer	5906 independent reflections
Radiation source: fine-focus sealed tube	4215 reflections with *I* > 2σ(*I*)
Graphite monochromator	*R*_int_ = 0.076
φ + ω scans	θ_max_ = 25.6°, θ_min_ = 1.6°
Absorption correction: multi-scan (MULscanABS in *PLATON*; Spek, 2009)	*h* = −12→12
*T*_min_ = 0.973, *T*_max_ = 1.000	*k* = −14→14
32665 measured reflections	*l* = −30→30

### Refinement

**Table:**

Refinement on *F*^2^	Primary atom site location: structure-invariant direct methods
Least-squares matrix: full	Secondary atom site location: difference Fourier map
*R*[*F*^2^ > 2σ(*F*^2^)] = 0.053	Hydrogen site location: inferred from neighbouring sites
*wR*(*F*^2^) = 0.102	H atoms treated by a mixture of independent and constrained refinement
*S* = 1.03	*w* = 1/[σ^2^(*F*_o_^2^) + (0.0461*P*)^2^ + 0.1416*P*] where *P* = (*F*_o_^2^ + 2*F*_c_^2^)/3
5906 reflections	(Δ/σ)_max_ < 0.001
381 parameters	Δρ_max_ = 0.24 e Å^−^^3^
1 restraint	Δρ_min_ = −0.34 e Å^−^^3^

### Special details

**Table:**

Geometry. Bond distances, angles *etc*. have been calculated using the rounded fractional coordinates. All su's are estimated from the variances of the (full) variance-covariance matrix. The cell e.s.d.'s are taken into account in the estimation of distances, angles and torsion angles
Refinement. Refinement of *F*^2^ against ALL reflections. The weighted *R*-factor *wR* and goodness of fit *S* are based on *F*^2^, conventional *R*-factors *R* are based on *F*, with *F* set to zero for negative *F*^2^. The threshold expression of *F*^2^ > σ(*F*^2^) is used only for calculating *R*-factors(gt) *etc*. and is not relevant to the choice of reflections for refinement. *R*-factors based on *F*^2^ are statistically about twice as large as those based on *F*, and *R*- factors based on ALL data will be even larger.

### Fractional atomic coordinates and isotropic or equivalent isotropic displacement parameters (Å^2^)

**Table:**

	*x*	*y*	*z*	*U*_iso_*/*U*_eq_	
Cl1	1.05031 (7)	0.59602 (6)	0.05700 (3)	0.0518 (3)	
O1	0.69721 (14)	0.42893 (12)	0.23989 (6)	0.0285 (5)	
N1	0.54054 (15)	0.36573 (13)	0.17599 (6)	0.0190 (5)	
N2	0.49236 (15)	0.04698 (13)	0.19441 (6)	0.0190 (5)	
N3	0.69523 (16)	−0.04818 (14)	0.14086 (7)	0.0205 (5)	
N4	0.65525 (16)	0.18840 (13)	0.09895 (6)	0.0196 (5)	
C1	0.45318 (19)	0.34058 (16)	0.21789 (8)	0.0211 (6)	
C2	0.47134 (19)	0.22457 (16)	0.24667 (8)	0.0211 (6)	
C3	0.41422 (19)	0.12539 (16)	0.21499 (8)	0.0201 (6)	
C4	0.41809 (19)	−0.03545 (16)	0.16818 (8)	0.0208 (6)	
C5	0.4779 (2)	−0.14266 (16)	0.15089 (8)	0.0223 (6)	
C6	0.59425 (19)	−0.12726 (16)	0.11840 (8)	0.0225 (6)	
C7	0.7886 (2)	−0.03802 (17)	0.10096 (8)	0.0224 (6)	
C8	0.86281 (19)	0.07432 (17)	0.10394 (8)	0.0233 (6)	
C9	0.77456 (19)	0.16747 (16)	0.08152 (8)	0.0211 (6)	
C10	0.59096 (19)	0.27254 (16)	0.06975 (8)	0.0204 (6)	
C11	0.45514 (19)	0.31136 (16)	0.07862 (8)	0.0212 (6)	
C12	0.46446 (19)	0.39816 (16)	0.12436 (8)	0.0219 (6)	
C13	0.3355 (2)	0.43797 (17)	0.14288 (9)	0.0275 (7)	
C14	0.3152 (2)	0.36450 (17)	0.19048 (9)	0.0255 (7)	
C15	0.3680 (2)	0.21172 (17)	0.08691 (9)	0.0252 (7)	
C16	0.3952 (2)	0.37268 (18)	0.02826 (9)	0.0291 (7)	
C17	0.6724 (2)	0.30665 (17)	0.03383 (9)	0.0270 (7)	
C18	0.7864 (2)	0.24086 (18)	0.04111 (9)	0.0279 (7)	
C19	0.9189 (2)	0.09679 (19)	0.16166 (9)	0.0278 (7)	
C20	0.9764 (2)	0.0633 (2)	0.07007 (10)	0.0352 (8)	
C21	0.7047 (2)	−0.06059 (18)	0.04720 (8)	0.0274 (7)	
C22	0.5666 (2)	−0.08356 (18)	0.06159 (8)	0.0259 (7)	
C23	0.5242 (2)	−0.20936 (17)	0.20181 (9)	0.0278 (7)	
C24	0.3731 (2)	−0.21047 (18)	0.11673 (9)	0.0304 (7)	
C25	0.2906 (2)	−0.00910 (17)	0.17232 (9)	0.0255 (7)	
C26	0.2880 (2)	0.09026 (17)	0.20192 (9)	0.0257 (7)	
C27	0.6151 (2)	0.20230 (17)	0.26588 (9)	0.0257 (7)	
C28	0.3973 (2)	0.23431 (19)	0.29592 (9)	0.0323 (8)	
C29	0.65598 (19)	0.41796 (16)	0.19249 (8)	0.0209 (6)	
C30	0.74298 (19)	0.45928 (16)	0.15280 (8)	0.0210 (6)	
C31	0.8608 (2)	0.40440 (18)	0.15117 (9)	0.0279 (7)	
C32	0.9543 (2)	0.44442 (19)	0.12113 (10)	0.0336 (7)	
C33	0.9312 (2)	0.54209 (19)	0.09329 (9)	0.0312 (7)	
C34	0.8160 (2)	0.59963 (18)	0.09487 (9)	0.0289 (7)	
C35	0.7220 (2)	0.55801 (16)	0.12428 (8)	0.0247 (7)	
H1	0.47190	0.39840	0.24610	0.0250*	
H2	0.57810	0.04950	0.19770	0.0230*	
H3N	0.7345 (19)	−0.0690 (17)	0.1721 (7)	0.0250*	
H4	0.62440	0.15290	0.12510	0.0230*	
H6	0.63730	−0.20130	0.11650	0.0270*	
H7	0.85420	−0.09910	0.10740	0.0270*	
H12	0.50810	0.46530	0.11140	0.0260*	
H13A	0.34150	0.51710	0.15350	0.0330*	
H13B	0.26240	0.42900	0.11410	0.0330*	
H14A	0.27030	0.29460	0.17860	0.0310*	
H14B	0.26260	0.40360	0.21490	0.0310*	
H15A	0.40200	0.17280	0.11960	0.0380*	
H15B	0.36690	0.16100	0.05660	0.0380*	
H15C	0.27900	0.23750	0.08980	0.0380*	
H16A	0.39670	0.32400	−0.00270	0.0440*	
H16B	0.44580	0.44000	0.02340	0.0440*	
H16C	0.30480	0.39310	0.03200	0.0440*	
H17	0.65510	0.36450	0.00850	0.0320*	
H18	0.85890	0.24660	0.02140	0.0330*	
H19A	0.96960	0.03230	0.17580	0.0420*	
H19B	0.84730	0.11020	0.18310	0.0420*	
H19C	0.97560	0.16230	0.16310	0.0420*	
H20A	0.94180	0.04710	0.03320	0.0530*	
H20B	1.03400	0.00270	0.08400	0.0530*	
H20C	1.02580	0.13290	0.07150	0.0530*	
H21A	0.70470	0.00480	0.02350	0.0330*	
H21B	0.73840	−0.12560	0.02920	0.0330*	
H22A	0.51370	−0.01480	0.06010	0.0310*	
H22B	0.52110	−0.13980	0.03760	0.0310*	
H23A	0.45140	−0.21860	0.22300	0.0420*	
H23B	0.59530	−0.16920	0.22280	0.0420*	
H23C	0.55540	−0.28250	0.19200	0.0420*	
H24A	0.33490	−0.16520	0.08670	0.0450*	
H24B	0.30480	−0.23240	0.13840	0.0450*	
H24C	0.41300	−0.27700	0.10330	0.0450*	
H25	0.21650	−0.05050	0.15780	0.0310*	
H26	0.21210	0.12650	0.21120	0.0310*	
H27A	0.64820	0.26010	0.29120	0.0380*	
H27B	0.66550	0.20300	0.23540	0.0380*	
H27C	0.62380	0.12960	0.28330	0.0380*	
H28A	0.43610	0.29370	0.31910	0.0480*	
H28B	0.30550	0.25160	0.28460	0.0480*	
H28C	0.40330	0.16380	0.31540	0.0480*	
H31	0.87760	0.33800	0.17110	0.0330*	
H32	1.03350	0.40500	0.11970	0.0400*	
H34	0.80130	0.66740	0.07590	0.0350*	
H35	0.64230	0.59720	0.12500	0.0300*	

### Atomic displacement parameters (Å^2^)

**Table:**

	*U*^11^	*U*^22^	*U*^33^	*U*^12^	*U*^13^	*U*^23^
Cl1	0.0417 (4)	0.0663 (5)	0.0499 (4)	−0.0137 (3)	0.0153 (3)	0.0165 (4)
O1	0.0300 (8)	0.0350 (9)	0.0190 (8)	−0.0049 (7)	−0.0032 (6)	−0.0026 (7)
N1	0.0205 (9)	0.0188 (8)	0.0168 (9)	0.0017 (7)	−0.0012 (7)	0.0010 (7)
N2	0.0145 (8)	0.0212 (8)	0.0213 (9)	−0.0005 (7)	0.0023 (7)	0.0011 (7)
N3	0.0221 (9)	0.0238 (9)	0.0150 (9)	0.0003 (7)	−0.0004 (7)	0.0019 (7)
N4	0.0221 (9)	0.0218 (9)	0.0147 (9)	−0.0017 (7)	0.0019 (7)	0.0051 (7)
C1	0.0199 (10)	0.0219 (10)	0.0217 (11)	0.0029 (8)	0.0035 (8)	−0.0018 (9)
C2	0.0223 (10)	0.0223 (10)	0.0191 (11)	0.0030 (8)	0.0039 (8)	−0.0001 (9)
C3	0.0211 (10)	0.0215 (10)	0.0183 (11)	0.0031 (8)	0.0046 (8)	0.0051 (8)
C4	0.0221 (11)	0.0219 (10)	0.0180 (11)	−0.0027 (8)	0.0010 (8)	0.0045 (9)
C5	0.0267 (11)	0.0180 (10)	0.0218 (11)	−0.0041 (8)	0.0012 (9)	−0.0005 (9)
C6	0.0254 (11)	0.0178 (10)	0.0237 (12)	0.0016 (8)	0.0002 (9)	−0.0022 (9)
C7	0.0237 (11)	0.0229 (10)	0.0211 (11)	0.0056 (9)	0.0050 (9)	−0.0009 (9)
C8	0.0196 (10)	0.0283 (11)	0.0223 (11)	0.0005 (9)	0.0039 (8)	0.0011 (9)
C9	0.0203 (10)	0.0230 (10)	0.0202 (11)	−0.0035 (8)	0.0029 (8)	−0.0005 (9)
C10	0.0240 (11)	0.0196 (10)	0.0163 (11)	−0.0036 (8)	−0.0024 (8)	0.0000 (8)
C11	0.0219 (11)	0.0226 (10)	0.0177 (11)	−0.0021 (8)	−0.0034 (8)	0.0032 (9)
C12	0.0229 (11)	0.0190 (10)	0.0223 (11)	0.0000 (8)	−0.0038 (8)	0.0051 (9)
C13	0.0247 (11)	0.0231 (11)	0.0340 (13)	0.0049 (9)	0.0001 (9)	0.0020 (10)
C14	0.0240 (11)	0.0245 (11)	0.0283 (12)	0.0065 (9)	0.0048 (9)	0.0025 (9)
C15	0.0237 (11)	0.0263 (11)	0.0240 (12)	−0.0035 (9)	−0.0033 (9)	0.0011 (9)
C16	0.0293 (12)	0.0318 (12)	0.0243 (12)	0.0007 (10)	−0.0048 (9)	0.0064 (10)
C17	0.0330 (12)	0.0270 (12)	0.0207 (11)	−0.0028 (9)	0.0021 (9)	0.0059 (9)
C18	0.0290 (12)	0.0335 (12)	0.0224 (12)	−0.0042 (10)	0.0087 (9)	0.0031 (10)
C19	0.0218 (11)	0.0307 (12)	0.0297 (13)	0.0003 (9)	−0.0017 (9)	0.0026 (10)
C20	0.0263 (12)	0.0431 (14)	0.0379 (14)	0.0040 (10)	0.0105 (10)	0.0044 (11)
C21	0.0325 (12)	0.0279 (12)	0.0216 (12)	−0.0034 (9)	0.0030 (9)	−0.0039 (9)
C22	0.0295 (12)	0.0254 (11)	0.0220 (12)	−0.0016 (9)	−0.0005 (9)	−0.0029 (9)
C23	0.0314 (12)	0.0230 (11)	0.0295 (13)	0.0012 (9)	0.0052 (10)	0.0036 (10)
C24	0.0311 (12)	0.0295 (11)	0.0306 (13)	−0.0083 (10)	0.0043 (10)	−0.0036 (10)
C25	0.0200 (11)	0.0250 (11)	0.0305 (13)	−0.0047 (9)	−0.0015 (9)	0.0072 (10)
C26	0.0203 (11)	0.0252 (11)	0.0322 (13)	0.0024 (9)	0.0062 (9)	0.0067 (10)
C27	0.0289 (12)	0.0209 (10)	0.0252 (12)	0.0027 (9)	−0.0050 (9)	−0.0001 (9)
C28	0.0420 (14)	0.0317 (12)	0.0249 (13)	0.0069 (10)	0.0107 (10)	0.0024 (10)
C29	0.0238 (11)	0.0158 (10)	0.0222 (12)	0.0029 (8)	−0.0006 (9)	−0.0015 (9)
C30	0.0225 (11)	0.0204 (10)	0.0187 (11)	−0.0049 (8)	−0.0037 (8)	−0.0045 (9)
C31	0.0240 (11)	0.0252 (11)	0.0336 (13)	0.0000 (9)	−0.0003 (9)	0.0033 (10)
C32	0.0233 (11)	0.0354 (13)	0.0422 (14)	0.0033 (10)	0.0038 (10)	0.0017 (11)
C33	0.0285 (12)	0.0400 (13)	0.0252 (12)	−0.0121 (10)	0.0042 (9)	0.0000 (11)
C34	0.0347 (13)	0.0263 (11)	0.0235 (12)	−0.0074 (10)	−0.0058 (9)	0.0037 (10)
C35	0.0257 (11)	0.0217 (11)	0.0251 (12)	0.0006 (9)	−0.0035 (9)	−0.0015 (9)

### Geometric parameters (Å, º)

**Table:**

Cl1—C33	1.745 (2)	C33—C34	1.381 (3)
O1—C29	1.233 (3)	C34—C35	1.385 (3)
N1—C1	1.503 (3)	C1—H1	1.0000
N1—C12	1.496 (3)	C6—H6	1.0000
N1—C29	1.368 (3)	C7—H7	1.0000
N2—C3	1.383 (3)	C12—H12	1.0000
N2—C4	1.376 (2)	C13—H13A	0.9900
N3—C6	1.475 (3)	C13—H13B	0.9900
N3—C7	1.482 (3)	C14—H14A	0.9900
N4—C9	1.380 (3)	C14—H14B	0.9900
N4—C10	1.378 (3)	C15—H15A	0.9800
N2—H2	0.8800	C15—H15B	0.9800
N3—H3N	0.882 (18)	C15—H15C	0.9800
N4—H4	0.8800	C16—H16A	0.9800
C1—C2	1.576 (3)	C16—H16B	0.9800
C1—C14	1.536 (3)	C16—H16C	0.9800
C2—C3	1.518 (3)	C17—H17	0.9500
C2—C27	1.530 (3)	C18—H18	0.9500
C2—C28	1.543 (3)	C19—H19A	0.9800
C3—C26	1.373 (3)	C19—H19B	0.9800
C4—C5	1.518 (3)	C19—H19C	0.9800
C4—C25	1.370 (3)	C20—H20A	0.9800
C5—C23	1.546 (3)	C20—H20B	0.9800
C5—C6	1.545 (3)	C20—H20C	0.9800
C5—C24	1.538 (3)	C21—H21A	0.9900
C6—C22	1.527 (3)	C21—H21B	0.9900
C7—C8	1.552 (3)	C22—H22A	0.9900
C7—C21	1.549 (3)	C22—H22B	0.9900
C8—C20	1.538 (3)	C23—H23A	0.9800
C8—C9	1.512 (3)	C23—H23B	0.9800
C8—C19	1.532 (3)	C23—H23C	0.9800
C9—C18	1.369 (3)	C24—H24A	0.9800
C10—C11	1.520 (3)	C24—H24B	0.9800
C10—C17	1.373 (3)	C24—H24C	0.9800
C11—C16	1.539 (3)	C25—H25	0.9500
C11—C12	1.555 (3)	C26—H26	0.9500
C11—C15	1.529 (3)	C27—H27A	0.9800
C12—C13	1.539 (3)	C27—H27B	0.9800
C13—C14	1.530 (3)	C27—H27C	0.9800
C17—C18	1.412 (3)	C28—H28A	0.9800
C21—C22	1.538 (3)	C28—H28B	0.9800
C25—C26	1.414 (3)	C28—H28C	0.9800
C29—C30	1.510 (3)	C31—H31	0.9500
C30—C35	1.395 (3)	C32—H32	0.9500
C30—C31	1.389 (3)	C34—H34	0.9500
C31—C32	1.384 (3)	C35—H35	0.9500
C32—C33	1.377 (3)		
			
C1—N1—C12	111.93 (15)	C21—C7—H7	108.00
C1—N1—C29	116.78 (15)	N1—C12—H12	107.00
C12—N1—C29	119.69 (15)	C11—C12—H12	107.00
C3—N2—C4	110.96 (16)	C13—C12—H12	107.00
C6—N3—C7	106.09 (15)	C12—C13—H13A	111.00
C9—N4—C10	110.73 (16)	C12—C13—H13B	111.00
C4—N2—H2	125.00	C14—C13—H13A	111.00
C3—N2—H2	124.00	C14—C13—H13B	111.00
C6—N3—H3N	113.1 (13)	H13A—C13—H13B	109.00
C7—N3—H3N	111.6 (13)	C1—C14—H14A	111.00
C10—N4—H4	125.00	C1—C14—H14B	111.00
C9—N4—H4	125.00	C13—C14—H14A	111.00
N1—C1—C14	104.17 (16)	C13—C14—H14B	111.00
N1—C1—C2	117.18 (16)	H14A—C14—H14B	109.00
C2—C1—C14	115.36 (16)	C11—C15—H15A	109.00
C1—C2—C28	105.28 (16)	C11—C15—H15B	109.00
C1—C2—C3	115.78 (16)	C11—C15—H15C	109.00
C1—C2—C27	111.11 (16)	H15A—C15—H15B	110.00
C27—C2—C28	108.11 (17)	H15A—C15—H15C	109.00
C3—C2—C27	109.37 (16)	H15B—C15—H15C	109.00
C3—C2—C28	106.78 (16)	C11—C16—H16A	109.00
C2—C3—C26	131.91 (18)	C11—C16—H16B	109.00
N2—C3—C26	106.15 (17)	C11—C16—H16C	110.00
N2—C3—C2	121.91 (17)	H16A—C16—H16B	110.00
C5—C4—C25	130.28 (18)	H16A—C16—H16C	109.00
N2—C4—C5	122.03 (17)	H16B—C16—H16C	109.00
N2—C4—C25	106.35 (17)	C10—C17—H17	126.00
C4—C5—C6	114.84 (16)	C18—C17—H17	126.00
C6—C5—C23	108.88 (16)	C9—C18—H18	126.00
C4—C5—C23	107.28 (16)	C17—C18—H18	126.00
C4—C5—C24	109.32 (17)	C8—C19—H19A	110.00
C6—C5—C24	107.66 (16)	C8—C19—H19B	109.00
C23—C5—C24	108.73 (16)	C8—C19—H19C	110.00
N3—C6—C22	100.71 (16)	H19A—C19—H19B	109.00
N3—C6—C5	115.48 (16)	H19A—C19—H19C	109.00
C5—C6—C22	118.22 (17)	H19B—C19—H19C	109.00
N3—C7—C8	113.32 (16)	C8—C20—H20A	109.00
N3—C7—C21	104.13 (16)	C8—C20—H20B	109.00
C8—C7—C21	114.65 (17)	C8—C20—H20C	109.00
C7—C8—C20	107.59 (17)	H20A—C20—H20B	109.00
C9—C8—C19	111.74 (17)	H20A—C20—H20C	110.00
C7—C8—C9	110.86 (16)	H20B—C20—H20C	109.00
C7—C8—C19	109.21 (17)	C7—C21—H21A	111.00
C9—C8—C20	108.63 (17)	C7—C21—H21B	111.00
C19—C8—C20	108.70 (17)	C22—C21—H21A	111.00
N4—C9—C8	122.41 (17)	C22—C21—H21B	111.00
N4—C9—C18	106.47 (17)	H21A—C21—H21B	109.00
C8—C9—C18	131.03 (18)	C6—C22—H22A	111.00
N4—C10—C17	106.38 (17)	C6—C22—H22B	111.00
N4—C10—C11	122.76 (17)	C21—C22—H22A	111.00
C11—C10—C17	130.85 (18)	C21—C22—H22B	111.00
C12—C11—C16	106.18 (16)	H22A—C22—H22B	109.00
C10—C11—C15	110.31 (16)	C5—C23—H23A	110.00
C15—C11—C16	107.75 (17)	C5—C23—H23B	109.00
C10—C11—C12	109.88 (16)	C5—C23—H23C	109.00
C10—C11—C16	108.21 (16)	H23A—C23—H23B	109.00
C12—C11—C15	114.24 (17)	H23A—C23—H23C	109.00
N1—C12—C13	101.44 (16)	H23B—C23—H23C	109.00
N1—C12—C11	117.13 (16)	C5—C24—H24A	109.00
C11—C12—C13	117.19 (17)	C5—C24—H24B	109.00
C12—C13—C14	105.32 (16)	C5—C24—H24C	109.00
C1—C14—C13	104.98 (16)	H24A—C24—H24B	110.00
C10—C17—C18	108.17 (19)	H24A—C24—H24C	109.00
C9—C18—C17	108.24 (19)	H24B—C24—H24C	110.00
C7—C21—C22	105.11 (16)	C4—C25—H25	126.00
C6—C22—C21	102.14 (16)	C26—C25—H25	126.00
C4—C25—C26	108.38 (18)	C3—C26—H26	126.00
C3—C26—C25	108.16 (18)	C25—C26—H26	126.00
O1—C29—N1	122.32 (18)	C2—C27—H27A	109.00
N1—C29—C30	120.88 (17)	C2—C27—H27B	109.00
O1—C29—C30	116.71 (17)	C2—C27—H27C	109.00
C31—C30—C35	118.22 (19)	H27A—C27—H27B	109.00
C29—C30—C35	123.90 (18)	H27A—C27—H27C	109.00
C29—C30—C31	117.07 (18)	H27B—C27—H27C	109.00
C30—C31—C32	121.4 (2)	C2—C28—H28A	109.00
C31—C32—C33	119.3 (2)	C2—C28—H28B	110.00
Cl1—C33—C32	119.82 (17)	C2—C28—H28C	109.00
Cl1—C33—C34	119.44 (17)	H28A—C28—H28B	109.00
C32—C33—C34	120.7 (2)	H28A—C28—H28C	109.00
C33—C34—C35	119.6 (2)	H28B—C28—H28C	109.00
C30—C35—C34	120.75 (19)	C30—C31—H31	119.00
N1—C1—H1	106.00	C32—C31—H31	119.00
C2—C1—H1	106.00	C31—C32—H32	120.00
C14—C1—H1	106.00	C33—C32—H32	120.00
N3—C6—H6	107.00	C33—C34—H34	120.00
C5—C6—H6	107.00	C35—C34—H34	120.00
C22—C6—H6	107.00	C30—C35—H35	120.00
N3—C7—H7	108.00	C34—C35—H35	120.00
C8—C7—H7	108.00		
			
C12—N1—C1—C2	126.13 (17)	C24—C5—C6—C22	50.7 (2)
C12—N1—C1—C14	−2.6 (2)	N3—C6—C22—C21	43.55 (18)
C29—N1—C1—C2	−90.6 (2)	C5—C6—C22—C21	170.30 (17)
C29—N1—C1—C14	140.62 (17)	N3—C7—C8—C9	−74.1 (2)
C1—N1—C12—C11	−106.27 (18)	N3—C7—C8—C19	49.4 (2)
C1—N1—C12—C13	22.59 (19)	N3—C7—C8—C20	167.26 (17)
C29—N1—C12—C11	111.7 (2)	C21—C7—C8—C9	45.3 (2)
C29—N1—C12—C13	−119.47 (18)	C21—C7—C8—C19	168.80 (17)
C1—N1—C29—O1	11.0 (3)	C21—C7—C8—C20	−73.4 (2)
C1—N1—C29—C30	−172.54 (16)	N3—C7—C21—C22	−0.6 (2)
C12—N1—C29—O1	151.33 (18)	C8—C7—C21—C22	−124.95 (18)
C12—N1—C29—C30	−32.3 (3)	C7—C8—C9—N4	53.4 (2)
C4—N2—C3—C2	178.98 (17)	C7—C8—C9—C18	−122.6 (2)
C4—N2—C3—C26	0.8 (2)	C19—C8—C9—N4	−68.7 (2)
C3—N2—C4—C5	−168.17 (18)	C19—C8—C9—C18	115.3 (2)
C3—N2—C4—C25	−0.2 (2)	C20—C8—C9—N4	171.42 (18)
C7—N3—C6—C5	−174.32 (16)	C20—C8—C9—C18	−4.6 (3)
C7—N3—C6—C22	−45.77 (18)	N4—C9—C18—C17	−0.3 (2)
C6—N3—C7—C8	154.10 (16)	C8—C9—C18—C17	176.2 (2)
C6—N3—C7—C21	28.87 (19)	N4—C10—C11—C12	84.7 (2)
C10—N4—C9—C8	−176.00 (18)	N4—C10—C11—C15	−42.2 (3)
C10—N4—C9—C18	0.9 (2)	N4—C10—C11—C16	−159.80 (18)
C9—N4—C10—C11	177.92 (17)	C17—C10—C11—C12	−96.6 (3)
C9—N4—C10—C17	−1.1 (2)	C17—C10—C11—C15	136.6 (2)
N1—C1—C2—C3	−76.6 (2)	C17—C10—C11—C16	18.9 (3)
N1—C1—C2—C27	48.9 (2)	N4—C10—C17—C18	0.9 (2)
N1—C1—C2—C28	165.73 (16)	C11—C10—C17—C18	−178.0 (2)
C14—C1—C2—C3	46.6 (2)	C10—C11—C12—N1	−53.0 (2)
C14—C1—C2—C27	172.15 (17)	C10—C11—C12—C13	−173.86 (17)
C14—C1—C2—C28	−71.0 (2)	C15—C11—C12—N1	71.6 (2)
N1—C1—C14—C13	−18.9 (2)	C15—C11—C12—C13	−49.3 (2)
C2—C1—C14—C13	−148.75 (17)	C16—C11—C12—N1	−169.75 (16)
C1—C2—C3—N2	108.6 (2)	C16—C11—C12—C13	69.3 (2)
C1—C2—C3—C26	−73.8 (3)	N1—C12—C13—C14	−33.75 (19)
C27—C2—C3—N2	−17.9 (3)	C11—C12—C13—C14	95.1 (2)
C27—C2—C3—C26	159.7 (2)	C12—C13—C14—C1	33.4 (2)
C28—C2—C3—N2	−134.63 (19)	C10—C17—C18—C9	−0.3 (3)
C28—C2—C3—C26	43.0 (3)	C7—C21—C22—C6	−26.4 (2)
N2—C3—C26—C25	−1.1 (2)	C4—C25—C26—C3	1.1 (3)
C2—C3—C26—C25	−179.0 (2)	O1—C29—C30—C31	65.7 (3)
N2—C4—C5—C6	−51.8 (3)	O1—C29—C30—C35	−103.8 (2)
N2—C4—C5—C23	69.4 (2)	N1—C29—C30—C31	−110.9 (2)
N2—C4—C5—C24	−172.90 (18)	N1—C29—C30—C35	79.6 (3)
C25—C4—C5—C6	143.4 (2)	C29—C30—C31—C32	−171.7 (2)
C25—C4—C5—C23	−95.5 (3)	C35—C30—C31—C32	−1.6 (3)
C25—C4—C5—C24	22.3 (3)	C29—C30—C35—C34	169.84 (19)
N2—C4—C25—C26	−0.5 (2)	C31—C30—C35—C34	0.4 (3)
C5—C4—C25—C26	166.1 (2)	C30—C31—C32—C33	1.6 (3)
C4—C5—C6—N3	48.0 (2)	C31—C32—C33—Cl1	177.89 (18)
C4—C5—C6—C22	−71.3 (2)	C31—C32—C33—C34	−0.4 (3)
C23—C5—C6—N3	−72.3 (2)	Cl1—C33—C34—C35	−179.02 (17)
C23—C5—C6—C22	168.44 (17)	C32—C33—C34—C35	−0.8 (3)
C24—C5—C6—N3	170.03 (16)	C33—C34—C35—C30	0.7 (3)

### Hydrogen-bond geometry (Å, º)

*Cg*1 is the centroid of pyrrole ring N2/C3/C4/C25/C26; *Cg*2 is the centroid of the benzene ring C30–C35.

**Table:**

*D*—H···*A*	*D*—H	H···*A*	*D*···*A*	*D*—H···*A*
N2—H2···N3	0.88	2.31	2.865 (2)	121
N4—H4···N3	0.88	2.55	3.051 (2)	117
C15—H15*A*···N2	0.98	2.52	3.488 (3)	171
C15—H15*A*···*Cg*1	0.98	2.40	3.301 (2)	152
N3—H3*N*···O1^i^	0.882 (18)	2.257 (18)	3.105 (2)	161.1 (18)
C23—H23*B*···O1^i^	0.98	2.53	3.495 (3)	168
C27—H27*C*···*Cg*2^i^	0.98	2.82	3.702 (2)	150

Symmetry code: (i) −*x*+3/2, *y*−1/2, −*z*+1/2.
